# Anemia correction using Roxadustat among MHD patients: 50mg versus 100mg dosing schedules

**DOI:** 10.6026/973206300221606

**Published:** 2026-03-31

**Authors:** Alam K.S, Masud Iqbal M, Rokhsana Alam Ripa, Shamsun Naher K.U.M, Babrul Alam Md

**Affiliations:** 1 Department of Nephrology, National Institute of Kidney Disease & Urology, Dhaka, Bangladesh

**Keywords:** Roxadustat, kidney disease, renal anemia, maintenance hemodialysis (MHD)

## Abstract

Anemia is a common complication in maintenance hemodialysis (MHD) patients. Hence, roxadustat, a hypoxia-inducible factor prolyl hydroxylase inhibitor, was evaluated at two dosing schedules (50 mg versus 100 mg) in a randomized, open-label study of 65 patients. Both doses significantly increased hemoglobin and reduced red cell transfusion requirements over 12 weeks. The 100 mg dose produced a quicker and more pronounced response. Headaches were frequent but manageable and blood pressure remained stable. Roxadustat was well tolerated, supporting its role as a viable treatment option for anemia in dialysis patients.

## Background:

Chronic kidney disease (CKD) is an escalating global health issue, marked by increasing incidence and prevalence rates. Renal anemia, a frequent complication of CKD, heightens the risk of morbidity and mortality, primarily due to the deficiency of endogenous erythropoietin (EPO) resulting from impaired kidney function. Additionally, inflammatory cytokines produced in damaged kidneys contribute to the decreased production of endogenous EPO [1, 2 and 3]. While erythropoiesis-stimulating agents (ESAs) can offer immediate benefits by increasing extremely low hemoglobin (Hb) levels and reducing the need for blood transfusions, evidence suggests that ESAs also raise the risk of stroke, hypertension, cardiovascular events and death. Additionally, ESAs must be stored under strict cold conditions during transportation and storage and some patients may develop resistance to these agents. Given these limitations, there is a pressing need for new and effective treatments for anemia in CKD patients [4]. Hypoxia- inducible factors (HIFs) are crucial transcriptional control complexes in the body's oxygen-sensing system, composed of a α and β subunit and tightly regulated by prolyl-hydroxyl domain (PHD) enzymes. Roxadustat, also known as FG-4592, is an oral HIF-prolyl- hydroxylase inhibitor (HIF-PHI) that mimics moderate hypoxia, stabilizes the HIF-α subunit, stimulates the production of endogenous EPO and enhances iron metabolism [5]. As HIF stabilizers, HIF- PHIs can stimulate the production of endogenous EPO and enhance iron mobilization and utilization by replicating the body's natural response to hypoxia. Several studies have demonstrated that Roxadustat effectively corrects anemia in dialysis patients [6, 7- 8]. Combining Roxadustat with ESA treatment could be more beneficial for improving renal anemia in MHD patients who exhibit ESA resistance, particularly during the first month of transitioning to Roxadustat therapy. The effects of Roxadustat on serum lipid levels have not yet been explored. Clarifying these effects is particularly important, as chronic HD patients typically have lower plasma high-density lipoprotein (HDL) - cholesterol levels compared to healthy individuals [9]. Therefore, it is of interest to report the efficacy and safety of Roxadustat in correcting anemia among MHD patients using two different dosing schedules (50 mg versus 100 mg).

## Materials and Methods:

This randomized, open-label, parallel-group study aimed to compare the efficacy of two different dosing schedules of Roxadustat (50 mg versus 100 mg, orally three times a week) in the correction of anemia among maintenance hemodialysis (MHD) patients who were dependent on red cell transfusions (RCTs). Conducted in the Sandors Dialysis Unit of National Institute of Kidney Diseases and Urology, Bangladesh, the study included MHD patients who relied solely on blood transfusions for anemia correction and excluded patients with blood pressure >160/100 mmHg despite four antihypertensive drugs, hemoglobin <7 g/dL, hemodialysis frequency <2 sessions per week, symptomatic heart failure, infections, active bleeding, thrombotic events, chronic liver disease, or malignancy. Participants were divided into two groups: Group 1 received 50 mg Roxadustat and Group 2 received 100 mg, both orally three times a week. The study spanned three months per patient to observe the hemoglobin response. Monthly follow-ups assessed the desired hemoglobin rise of 0.5-1 g/dL. Clinical and laboratory tests, including complete blood count (CBC), urea, creatinine, total protein, albumin, lipid profile, uric acid and ferritin levels, were conducted at baseline and during monthly follow-ups. Initially, 84 MHD patients were enrolled, with 65 included in the final analysis. Baseline characteristics such as age, BMI, hemoglobin, ferritin and post-dialysis urea reduction were comparable between groups. Hemoglobin changes over 0, 4, 8 and 12 weeks were recorded, showing significant increases in the 100 mg group compared to the 50 mg group. The study found that RCT requirements reduced significantly during the trial, with a notable decrease from 1.1±0.3 units/month at the start to 0.5±0.2 units/month at the end. Ethical clearance for this study was obtained from the IRB of NIKDU under memo no: NIKDU/ERC/2023/128(a). The study also received support from SK & F for research grants and study drugs and KDRG for logistical and scientific collaboration.

## Results:

The mean age of participants was 44 ± 11 years in the 50 mg group and 46 ± 13 years in the 100 mg group. The mean Body Mass Index (BMI) was slightly higher in the 100 mg group (23 ± 5 kg/m^2^) compared to the 50 mg group (21 ± 1 kg/m^2^). Baseline hemoglobin levels were similar between the groups, with the 50 mg group having 8.9 ± 1.5 g/dL and the 100 mg group having 8.8 ± 1.2 g/dL. Ferritin levels were also comparable, with the 50 mg group at 1416 ± 977 µg/L and the 100 mg group at 1610 ± 780 µg/L. The urea reduction percentages were identical in both groups, with a mean of 65 ± 17% in the 50 mg group and 65 ± 12% in the 100 mg group (Table 1 - see PDF). The study observed hemoglobin changes over a 12-week period in both the 50 mg and 100 mg Roxadustat groups. At baseline, the hemoglobin levels were similar between the groups, with the 50 mg group having 8.9 ± 1.6 g/dL and the 100 mg group having 8.8 ± 1.2 g/dL, with no significant difference (NS) between or within groups. At the 4-week mark, the 50 mg group had a hemoglobin level of 8.8 ± 1.6 g/dL, while the 100 mg group showed an increase to 9.3 ± 1.5 g/dL. The within-group changes were not significant for either group; however, the between-group comparison showed a significant difference (p < 0.05). By 8 weeks, the 50 mg group had a hemoglobin level of 9.1 ± 1.3 g/dL and the 100 mg group increased to 9.5 ± 2.3 g/dL. Again, the within-group changes were not significant, but the difference between groups remained significant (p < 0.05). At the end of 12 weeks, the hemoglobin level in the 50 mg group was 9.1 ± 1.5 g/dL, whereas the 100 mg group had a level of 9.5 ± 1.5 g/dL. The within-group changes were still not significant, but the difference between groups continued to be significant (p < 0.05) (Table 2 - see PDF). At baseline, the 50 mg group required 1.1 ± 0.3 units of blood per month, while the 100 mg group required 1.2 ± 0.1 units per month, with no significant difference between the groups (NS). After 12 weeks of treatment, both groups showed a significant reduction in blood transfusion requirements. The 50 mg group reduced to 0.5 ± 0.2 units per month and the 100 mg group similarly reduced to 0.5 ± 0.2 units per month. The within-group changes were significant for both groups (p < 0.025) (Table 3 - see PDF). In the 50 mg group, 27 out of 32 participants (84.4%) experienced headaches, while 5 participants (15.6%) reported no headaches. Similarly, in the 100 mg group, 29 out of 33 participants (87.9%) experienced headaches and 4 participants (12.1%) did not report headaches (Table 4 - see PDF). Blood pressure and antihypertensive drug use were monitored and compared between the two groups over the study period. At baseline, the systolic blood pressure (SBP) in the 50 mg group was 138.1 ± 20.4 mmHg, while the 100 mg group had an SBP of 141.5 ± 19.2 mmHg, with no significant difference (NS) between the groups. The diastolic blood pressure (DBP) at baseline was 84.1 ± 7.6 mmHg in the 50 mg group and 82.1 ± 10.5 mmHg in the 100 mg group, also showing no significant difference (NS). At the 12-week mark, the SBP in the 50 mg group increased to 150.9 ± 20.7 mmHg, whereas the 100 mg group had a slightly lower SBP of 142.7 ± 17.7 mmHg, with no significant difference (NS) between the groups. The DBP at 12 weeks was 83.8 ± 7.1 mmHg in the 50 mg group and 83.3 ± 8.9 mmHg in the 100 mg group, with no significant difference (NS). Regarding antihypertensive drug use, the 50 mg group used an average of 2.2 ± 1.2 drugs at baseline, which remained the same at 12 weeks. The 100 mg group used an average of 1.8 ± 1.3 drugs at baseline and maintained this usage at 12 weeks, with no significant difference (NS) between the groups at either time point (Table 5 - see PDF). The study also evaluated the red cell transfusion (RCT) requirements at the start and end of the study for both the 50 mg and 100 mg groups. At the start of the study, the 50 mg group required 1.0 ± 0.4 units of blood per month, while the 100 mg group required 1.1 ± 0.1 units per month, with no significant difference (NS) between the groups. By the end of the 12-week study period, the RCT requirements had decreased in both groups. The 50 mg group required 0.8 ± 0.8 units per month and the 100 mg group required 0.6 ± 0.2 units per month. Despite these reductions, the difference between the two groups remained non-significant (NS) (Table 6 - see PDF).

## Discussion:

This study aimed to evaluate the efficacy and safety of two different dosing schedules of Roxadustat (50 mg versus 100 mg) in correcting anemia among maintenance hemodialysis (MHD) patients dependent on red cell transfusions (RCTs). Our findings indicate that both dosing schedules effectively increased hemoglobin levels and significantly reduced the need for blood transfusions, with the 100 mg dose showing a more pronounced and quicker response. At baseline, the mean age, BMI and hemoglobin levels of participants were comparable between the 50 mg and 100 mg groups. The baseline hemoglobin levels were consistent with other studies evaluating the efficacy of Roxadustat in CKD patients. For instance, Fishbane *et al.* reported baseline hemoglobin levels of around 9.1 g/dL in both treatment groups, indicating similar starting points for comparison [10]. This comparability ensures the robustness of our findings, aligning with existing literature and providing a solid foundation for evaluating treatment effects. The hemoglobin changes observed over the 12-week period demonstrated that the 100 mg dose of Roxadustat was more effective in increasing hemoglobin levels compared to the 50 mg dose. By the 4th week, the 100 mg group showed a significant increase in hemoglobin levels (9.3 ± 1.5 g/dL) compared to the 50 mg group (8.8 ± 1.6 g/dL), with the trend continuing through the 8th and 12th weeks. These results are consistent with the findings of Fishbane *et al.* who reported significant hemoglobin increases with Roxadustat in CKD patients not on dialysis, further supporting the efficacy of Roxadustat in managing anemia in this patient population [10]. Additionally, a study by Odajima *et al.* found that Roxadustat effectively increased hemoglobin levels in non-dialysis- dependent CKD patients, demonstrating the drug's potential across different CKD populations [8]. Regarding blood transfusion requirements, both dosing groups showed a significant reduction by the end of the 12-week period. The 50 mg group reduced from 1.1 ± 0.3 units/month to 0.5 ± 0.2 units/month and the 100 mg group from 1.2 ± 0.1 units/month to 0.5 ± 0.2 units/month. This significant reduction aligns with the results reported by Csiky *et al.* who found that Roxadustat effectively reduced the need for red blood cell transfusions in dialysis patients [11]. The reduction in transfusion requirements highlights the potential of Roxadustat to minimize the risks associated with frequent blood transfusions, such as iron overload and alloimmunization. Similarly, Provenzano *et al.* in their pooled analysis of phase 3 studies observed that Roxadustat significantly reduced transfusion requirements compared to placebo, reinforcing our findings [12]. The incidence of headaches as an adverse event was slightly higher in the 100 mg group (87.9%) compared to the 50 mg group (84.4%). This finding is comparable to the adverse event profiles reported in other studies. For example, Fishbane *et al.* noted headaches as a common adverse event, with similar frequencies across different dosing groups [10]. The consistent occurrence of headaches across studies suggests that this adverse effect is manageable and does not significantly detract from the overall benefits of Roxadustat therapy. Furthermore, Fishbane *et al.* reported a comparable incidence of headaches in their phase 3 studies, confirming the adverse event profile observed in our study [10]. Blood pressure and antihypertensive drug use remained stable throughout the study, with no significant differences observed between the two dosing groups. At baseline, the systolic and diastolic blood pressures were similar between the groups and this trend continued through the 12-week period. This stability in blood pressure is consistent with findings from Barratt *et al.* who reported that Roxadustat did not significantly impact mean arterial pressure or the incidence of hypertension in CKD patients [13]. Cheng *et al.* found that Roxadustat had a minimal impact on blood pressure compared to recombinant human erythropoietin, further supporting the safety of Roxadustat concerning blood pressure management in CKD patients [14]. Additionally, Liu *et al.* investigated the pharmacokinetics of Roxadustat and found no significant changes in blood pressure, corroborating the safety profile observed in our study [15]. In conclusion, this study demonstrates that Roxadustat, at both 50 mg and 100 mg dosing schedules, is effective in correcting anemia in MHD patients and significantly reducing the need for blood transfusions. The 100 mg dose provides a more significant and quicker response in increasing hemoglobin levels without disproportionately increasing adverse events. These findings are supported by and align with existing literature, further validating Roxadustat as a viable treatment option for anemia in CKD patients. Future studies should explore the long-term effects of different dosing schedules and their impact on overall patient outcomes.

## Limitations:

The study includes its conduction in a single hospital with a small sample size, which may limit the applicability of the results to the broader community. These findings suggest that Roxadustat is a viable and effective treatment option for anemia in CKD patients on dialysis, with a favorable safety profile. Future studies should focus on the long-term efficacy and safety of Roxadustat, as well as its impact on overall patient outcomes.

## Conclusion:

We show that Roxadustat, administered at both 50 mg and 100 mg dosing schedules, is effective in correcting anemia among maintenance hemodialysis (MHD) patients. Both dosing schedules significantly increased hemoglobin levels and reduced the need for blood transfusions over the 12-week period. The 100 mg dose showed a more pronounced and quicker response compared to the 50 mg dose. Adverse events, including headaches, were common but manageable and there were no significant changes in blood pressure or antihypertensive drug use in either group.
